# COVID-19 Communication Ecologies: Using Interpersonal, Organizational, and Mediated Communication Resources to Cope With a Pandemic

**DOI:** 10.1177/0002764221992837

**Published:** 2021-06

**Authors:** J. Brian Houston

**Affiliations:** 1 University of Missouri, Columbia, MO, USA

**Keywords:** communication ecology, COVID-19, public health emergency, crisis, coping

## Abstract

Information and communication resources are needed for individuals to cope with a public health emergency like the COVID-19 pandemic. These resources include interpersonal, organizational, and mediated communication, which collectively constitute a communication ecology. This interdisciplinary special issue of *American Behavioral Scientist* focuses on applications of a communication ecology perspective to the COVID-19 pandemic. Each article in this issue examines one or more specific aspect of COVID-19 communication ecologies to expand understanding of how a variety of communication resources can foster individual and collective coping with a global public health crisis. Insights from this issue can inform ongoing response to COVID-19 and planning for future public health crises.

As a public health emergency, the COVID-19 pandemic poses significant physical and mental health risks for individuals. In terms of physical health, as of January 2021 there have been over 82 million COVID-19 cases resulting in almost 2 million deaths (https://coronavirus.jhu.edu/map.html). Even individuals who survive COVID-19 may experience serious illness, with some reporting prolonged symptoms (Callard & Perego, 2021). The COVID-19 pandemic also has public mental health consequences, as individuals have experienced increased anxiety, depression, and grief reactions (Eisma & Tamminga, 2020; First et al., 2020). These mental health reactions are often related to knowing someone affected by COVID-19 (e.g., experiencing the death of a family member related to COVID-19), worrying about keeping oneself and family members safe, experiencing uncertainty about the severity of and susceptibility to the disease, attempting to cope with financial impacts resulting from the pandemic, and being disconnected from others as a consequence of isolation, quarantine, and lockdowns.

A variety of resources are needed for individuals to successfully cope with a public health emergency like the COVID-19 pandemic. Specifically, people need health care, economic, social welfare, and communication resources. In the case of COVID-19, health care resources include vaccines to prevent the disease and medical treatment for individuals who are ill. Economic resources include financial assistance and resources for people whose ability to work and earn money has been interrupted by the pandemic. Social welfare resources include assistance with food and housing for individuals affected by the pandemic and the continuation of education despite ongoing social lockdowns. Communication resources include sources of information and support than can help individuals gain information related to the pandemic and stay connected with others.

The current issue of *American Behavioral Scientist* focuses specifically on exploring the role of communication resources during the COVID-19 pandemic. In approaching this topic, contributing authors take a broad, *ecological* approach to communication resources. A communication ecology perspective considers interpersonal, organizational, and mediated communication resources. Insights from the research included in this issue can be utilized as the world continues to respond to the current pandemic, and can be applied to future public health emergencies and disasters. To begin, a brief overview of the concept of communication ecology is provided and applied to the COVID-19 pandemic.

## COVID-19 Communication Ecology

Communication ecologies are “the networks of communication connections that groups or individuals depend upon in order to achieve a goal” (Broad et al., 2013, p. 328). Thus, communication ecologies are goal-specific, so that different ecologies likely exist for varied purposes. Similar to conceptualizations of a disaster communication ecology (see, e.g., Spialek & Houston, 2019), individuals are likely to construct a COVID-19 communication ecology to meet the goal of coping with the threat and negative impact of the COVID-19 pandemic. Communication resources are included in a communication ecology to the extent that a resource provides utility in meeting the relevant goal (Ball-Rokeach, 1985).

**Figure 1.**
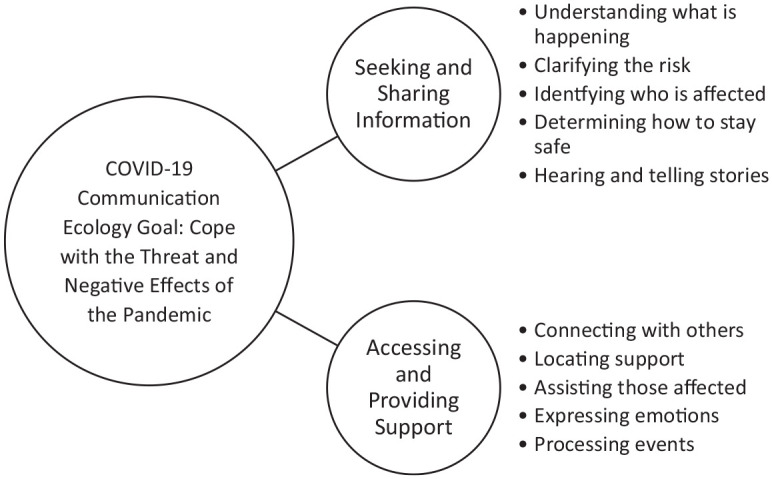
Goal and functions of a COVID-19 communication ecology.

Specifically, individuals may utilize a COVID-19 communication ecology to seek and share information about the pandemic and to gain and provide support (see Figure 1). In the modern communication and media environment, individuals can function as both users and producers of information within a communication ecology (Houston et al., 2015). For example, a person can use a social media site like Twitter to find out current information about the COVID-19 pandemic and can also post his or her own COVID-19 experience or opinions. Thus, individuals can take from and contribute to the communication ecology.

**Figure 2.**
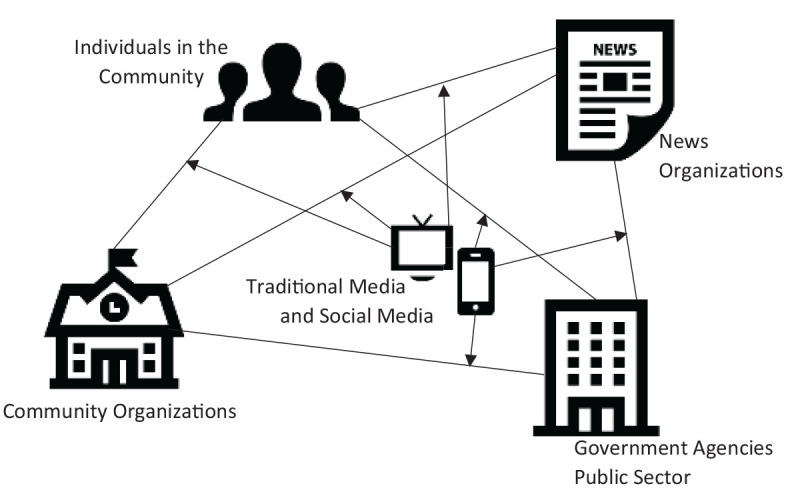
COVID-19 communication ecology. *Note*. In a community ecology, communication resources are potentially connected directly (via interpersonal interactions that occur in person) and through mediated interactions (via traditional or social media).

The communication resources potentially included in a communication ecology are multifaceted and can include interpersonal, organizational, and mediated sources (Ball-Rokeach et al., 2001; Okon, 2015: Wilkin et al., 2007). Thus, COVID-19 communication resources can include family and friends, local organizations, news organizations, and government agencies (see Figure 2). The utility of employing a communication ecology approach to understand how individuals use communication to achieve goals is that it provides a robust and realistic perspective on the ways that people often use a variety of diverse sources to gain the information and support they need. Most public health emergency communication research focuses on discrete communication messages (e.g., specific emergency warning messages) or individual communication forms (e.g., television, social media, organizational communication) to better understand communication during crises. While this work is useful and needed, it is also helpful to take broad views of the communicative environment to gain a comprehensive picture of human communication during collective crises. This broad communication view is the focus of the current special issue.

## The COVID-19 Communication Ecology Special Issue

This interdisciplinary special issue of *American Behavioral Scientist* focuses on applications of a communication ecology perspective to the COVID-19 pandemic. Houston et al. (this issue) begin the special issue with a survey of U.S. adults to examine the structure of a COVID-19 communication ecology using network analysis. In this article, the authors introduce the communication ecology network (CEN) model, which posits that similar communication resources will cluster together within a communication ecology. Their analysis identifies five communication clusters within the overall network, and they note that the most frequently used communication cluster (television news) was least integrated into the overall ecology. They also assess how communication resource use is associated with belief in COVID-19 misinformation and identify several important relationships.

Liu et al. (this issue) analyze social media communication of government and disaster management agencies in the U.S. state of Texas and find that the pattern of communication across the COVID-19 crisis is relatively consistent. Additionally, they find that state and federal agencies function as agenda setters within the communication ecology during the COVID-19 crisis. Tagliacozzo et al. (this issue) explore the communication of public health agencies in three countries (Italy, Sweden, the United States) during the early period of the COVID-19 pandemic. They find that the public health agencies relied on their own scientific expertise and coordinated primarily with other government agencies. Across countries, the authors found public health agency information to be lacking for many vulnerable groups (e.g., pregnant women, individuals with disabilities, immigrants, homeless populations).

Hernandez and Colaner (this issue) conducted interviews to understand how children manage chronic uncertainty about COVID-19 in communication with their parents. Their results indicate that children use family communication with parents to navigate uncertainty related to COVID-19, media, politics, and time. The authors also find that that families most often reference macro communication resources as important sources of information to help with COVID-19 uncertainty.

Perreault and Perreault (this issue) examine the role of journalists and news organizations during the COVID-19 pandemic. Journalists and news organizations function as both an important source of information during a public health emergency like COVID-19 and also draw from other resources in the communication ecology to inform their reporting. Perreault and Perreault interview journalists and find journalists’ connections with other communication resources in the ecology to be strained as a result of the health threats resulting from COVID-19 and the economic threats to journalism that predate the pandemic.

Cannon et al. (this issue) consider the specific stressors that are associated with the occurrence of intimate partner violence (IPV) during COVID-19. They find that income loss, nutritional stress, and housing characteristics are related to an increased prevalence of IPV during the pandemic. The authors discuss the communication resources needed to support individuals experiencing IPV given these results.

Each of these articles examine one or more specific aspects of COVID-19 communication ecologies to expand our understanding of how a variety of communication resources can foster individual and collective coping with a public health crisis like the COVID-19 pandemic.
